# Feasibility of a multicenter prospective cohort study on the evaluation of prognosis in severe traumatic brain injury

**DOI:** 10.1186/cc10917

**Published:** 2012-03-20

**Authors:** AF Turgeon, F Lauzier, M Thibodeau, A Rigamonti, M Meade, F Bernard, K Burns, K Reddy, D Scales, L McIntyre, R Green, D Griesdale, L Moore, M Savard, D Jichici, J Paquet, D Zygun, D Fergusson

**Affiliations:** 1 Université Laval, Québec, Canada; 2 University of Toronto, Ontario, Canada; 3 McMaster University, Ontario, Canada; 4 Université de Montréal, Québec, Canada; 5 University of Ottawa, Ontario, Canada; 6 University Dalhousie, Nova Scotia, Canada; 7 University of British Columbia, Canada; 8 University of Calgary, Alberta, Canada

## Introduction

Conducting prospective research in severe traumatic brain injury (TBI) patients is challenging. To prepare for a large-scale multicenter study to evaluate long-term prognosis in severe TBI, we conducted a prospective pilot study evaluating the patterns of enrollment, the compliance to the schedule of prognostic tests and the completeness of follow-up for 6-month functional outcome measures.

## Methods

We conducted a pilot study in nine level I trauma centers in Canada. Adult patients with severe TBI expected to require mechanical ventilation for ≥48 hours were enrolled on their first day in the ICU. Prognostic tests were performed on arrival (CT scan), day 1 (serum biomarker), day 3 (serum biomarker, CT scan) and day 7 (serum biomarker, CT scan, MRI, SSEP, EEG) with time windows of 24 or 48 hours depending on the test. Prognostic measures were collected during the first week in the ICU to examine the association with the extended Glasgow Outcome Scale score. We considered as appropriate a compliance to the schedule of prognostic tests ≥90% and a proportion of lost to follow-up <10%. We obtained REB approval from participating centers and written informed consent from SDMs.

## Results

Among 116 consecutive eligible patients, 50 were enrolled over a total of 204 weeks of screening between May 2010 and May 2011. Two centers used a deferred consent approach. Patients were primarily male with a median age of 45 years and a GCS of 5 (25th to 75th: 3 to 7). The two main reasons for nonenrollment were the time window for inclusion being after regular working hours (35%, *n *= 23) and oversight (24%, *n *= 16). The compliance to the different tests ranged from 93 (three missing tests) to 100%. All blood samples but one (day 7) were performed. The main reason for missing a test was the patient's instability (hemodynamic or increased ICP) (*n *= 5). In six patients, the MRI had to be delayed due to the presence of material not compatible with the procedure. No patient was lost to follow-up at 6 months.

## Conclusion

These results demonstrate the feasibility of enrollment and complying to a structured protocol of prognostic tests in a prospective multicenter study in severe TBI patients.

